# Tuberculosis, before and after Antiretroviral Therapy among HIV-Infected Children in Nigeria: What Are the Risk Factors?

**DOI:** 10.1371/journal.pone.0156177

**Published:** 2016-05-27

**Authors:** Emmanuel A. Anígilájé, Sunday A. Aderibigbe, Adekunle O. Adeoti, Nnamdi O. Nweke

**Affiliations:** 1 Department of Paediatrics, Federal Medical Centre, Makurdi, Benue State, Nigeria; 2 Department of Community Medicine, University of Ilorin, Ilorin, Kwara State, Nigeria; 3 Department of Medicine, Ekiti State University Teaching Hospital, Ado-Ekiti, Ekiti State, Nigeria; Aga Khan University Hospital Nairobi, KENYA

## Abstract

**Introduction:**

In Nigeria, there is a dearth of pediatric data on the risk factors associated with tuberculosis (TB), before and after antiretroviral therapy (ART).

**Methodology:**

A retrospective observational cohort study, between October 2010 and December 2013, at the Federal Medical Centre, Makurdi, Nigeria. TB was noted among children less than 15 years of age at ART enrolment (prevalent TB-PrevTB), within 6 months (early incident tuberculosis-EITB) and after 6 months (late incident tuberculosis-LITB) of a 12-month follow-up on ART. Potential risk factors for PrevTB and incident TB were assessed using the multivariate logistic and Cox regression models respectively.

**Results:**

Among 368 HIV-1 infected children, PrevTB was diagnosed in 73 children (19.8%). Twenty-eight EITB cases were diagnosed among 278 children over 132 person-years (py) with an EITB rate of 21.2/100 py. Twelve LITB cases were seen among 224 children over 221.9 py with a LITB rate of 5.4/100 py. A significant reduction in the incidence rates of TB was found over time (75%, p˂ 0.001). Young age of children (12–35 months, aOR; 24, 95% CI; 4.1–146.6, p ˂ 0.001; 36–59 months, aOR;21, 95%CI;4.0–114.3, p ˂ 0.001); history of TB in children (aOR; 29, 95% CI; 7.3–119.4, P˂ 0.001); severe immunosuppression (aOR;38, 95% CI;12–123.2,p ˂ 0.001); oropharyngeal candidiasis (aOR;3.3, 95% CI; 1.4–8.0, p = 0.009) and sepsis (aOR; 3.2, 95% CI;1.0–9.6, p = 0.043) increased the risk of PrevTB. Urban residency was protective against EITB (aHR; 0.1, 95% CI; 0.0–0.4, p = 0.001). Virological failure (aHR; 4.7, 95% CI; 1.3–16.5, p ˂ 0.001) and sepsis (aHR; 26, 95% CI; 5.3–131.9, p ˂ 0.001) increased the risk of LITB.

**Conclusions:**

In our cohort of HIV-infected children, a significant reduction in cases of incident TB was seen following a 12-month use of ART. After ART initiation, TB screening should be optimized among children of rural residency, children with sepsis, and those with poor virological response to ART.

## Introduction

In 2012, among children of the world, 530,000 became newly infected with tuberculosis (TB) [1] and 260, 000, with human immunodeficiency virus (HIV) [2]. Co-infection with both organisms is an increasing global emergency. Reported prevalence of HIV/TB co-infection in children ranges from <5% in industrialized settings, to over 50% in some African settings [3–7] and Nigeria ranks fifth among the 22 high TB burden countries [1].

Complex interactions also exist between HIV and TB. For example, HIV-infected children demonstrate greater mortality from TB, with mortality as high as 20–35% in resource limited settings [8–10]. Also, HIV-infected children have an increased risk of rapid TB disease progression [11], a higher likelihood of unsatisfactory response to TB treatment [7, 8–10, 12] and a higher risk of TB recurrence [8]. Whereas, antiretroviral therapy (ART) reduces TB incidence in HIV-infected children [9, 10, 13, 14], the incidence of TB still remains substantially higher in HIV positive children than in the general paediatric population [15]. Although, the mechanisms promoting the susceptibility of people with HIV to TB disease are incompletely understood, several multifactorial processes have been described [16].

Although the number of Nigerian children (aged 0–14 years) newly acquiring HIV infection decreased from 65,000 in 2009 to 59,000 in 2012; access to ART among those eligible increased meagerly from 8% to 12% [2]. As ART had been proven to be protective against the acquisition of TB among HIV-infected subjects [9, 10, 13, 14], it may be implied that a reasonable number of these children who did not have access to ART have been left unprotected against TB within this time frame. According to the World Health Organization (WHO) Global Tuberculosis Report of 2013, about 97, 853 cases of TB was notified in 2012 among Nigerian adults population, and about 1,187 new smear- positive cases of TB was reported in children, aged 0–14 years, the same year [1]. Furthermore, about 15.6% to 50% of HIV-infected Nigerian children enrolling into ART services have a current TB diagnosis at the time of starting ART [11, 12, 17–19].

In 1991, the Nigerian National Tuberculosis and Leprosy Control Programme (NTBLCP) was launched, with a mandate of coordinating TB and Leprosy Control activities in the country [20]. The goal of the NTBLCP was to reduce the burden of TB by 2015 and the targets were to reduce the prevalence (old and new TB cases) and death rates from TB by 50%, relative to the 1990 levels [20]. The NTBLCP is expected to control the acquisition of TB among people living with HIV/AIDS and also, to reduce HIV infection rate among TB patients [20]. The NBTLCP utilizes the STOP-TB control strategies [20]. However, the NTBLCP activities have been hampered by many challenges, which cumulatively reduce its successes [21, 22]. Some of these challenges include; poor funding, passive case finding for TB at ART programmes by screening only patients with symptoms of TB, the lack of the necessary diagnostic tools/expertise needed for early diagnosis of TB among HIV -infected persons, the lack of adequate knowledge on the limitations of the existing diagnostic tools, ineffective implementation of the Isoniazid Preventive Therapy (IPT), the poor expansion of the Directly Observed Treatment Short Course (DOTS) therapy, poor involvement of the private health facilities in TB/HIV care services and the placement of undue emphasis on smear- positive TB in children.

Also, the dearth of local studies to describe the risk factors of TB among patients on ART in ART programmes is another challenge that has reduced the success of the NTBLCP. At the time of this study and as far as we are aware, only the study of Akanbi *et al*. [23] in Jos had reported the risk factors of incident TB among Nigerian adult patients on ART. They reported poor immunologic and/or virologic response to ART to be the significant risk factors associated with incident TB [23]. However, the incidence rate and risk factors for TB co-infection after the initiation of ART in Nigerian children, is not known.

This retrospective cohort study therefore aims to determine: the prevalent TB (PrevTB) at ART enrolment, the respective early incident TB (EITB) and late incident TB (LITB) during the first 6 months and the later 6 months of ART follow-up, and the risk factors associated with both prevalent and incident TB among Nigerian HIV-infected children.

## Materials and Methods

### Study area and setting

The study was carried out at the Paediatric ART Clinic of the Riverside Specialist Clinics of the Federal Medical Centre (FMC), Makurdi, Benue State, Nigeria. FMC, Makurdi is a tertiary health institution owned and funded by the Federal Government of Nigeria. The facility is supported by the AIDS Prevention Initiative in Nigeria (APIN)/Harvard PEPFAR (The USA President’s Emergency Plan for AIDS Relief) programme in its care and treatment of HIV-infected patients. Comprehensive care for the HIV-infected paediatric patients started at the FMC, Makurdi, in May 2006. Details of the study setting have been reported elsewhere [24]. The paediatric ART clinic was manned by two paediatricians and 8 paediatric residents at the time of this study. The clinic is run twice weekly, on Wednesdays and Fridays. Between May 2006 and December 2013, the program had cumulatively recruited 1,278 children, of whom 834 had been initiated on ART. Children were recruited into care and treatment if they were confirmed to be HIV-1 infected. Infants less than 18 months old were regarded as being HIV-infected if two samples were positive for HIV DNA/PCR. For children ≥ 18 months, a positive HIV serology was confirmed by Western blot in children who had initial double rapid HIV antibody tests using Determine HIV 1 & 2 first and HIV 1& 2 STATPAK in serial algorithm. Scheduled follow-up of subjects was as follows: monthly for the first three months, every 3-month in the first year and thereafter, every 6-month. The growth parameters, the viral load, the CD4 counts and co-morbidity/opportunistic infection were determined at each scheduled and event triggered visit.

### Ethical consideration

Upon recruitment into care and the ART programme, parents or caregivers of the HIV-infected children had provided written informed consent for the use of their data for research as approved by the Research and Ethics Committee of the Federal Medical Centre, Makurdi, Benue State, Nigeria and the AIDS Prevention Initiative in Nigeria (APIN)/Harvard PEPFAR.

### Study design and population

This was a retrospective observational cohort study between October 2010 and December 2013. Included in the study were HIV-infected children (0–15 years) who were initiated on ART during the study period and whose relevant data (i.e., socio-demographic, clinical/laboratory, diagnosed co-morbidities/ opportunistic infections) were available. This included subjects who were diagnosed with TB at enrollment and those who developed TB, consequently on follow-up on ART for 1 year. Excluded were children on ART with missing relevant data of interest as described previously and subjects older than 15 years, who were routinely seen at the adult ART clinic of the FMC Makurdi.

### Patients follow-up

For all children, antiretroviral therapy was commenced in accordance with the clinical and age-dependent immunological criteria of World Health Organization (WHO) guidelines of 2006 and 2010 and as adopted in the document of the Nigerian *National Guidelines for Paediatric HIV and AIDS Treatment and Care* [25, 26]. Cotrimoxazole was also given to all subjects from the time of HIV diagnosis irrespective of their CD4 counts levels. Also, during the period of study, children less than 24 months with HIV/TB co- infection were commenced on ART, regardless of their CD4 counts levels. The first line ART regimen consisted of Zidovudine (AZT) or Stavudine (D4T) plus Lamivudine (3TC) plus Nevirapine (NVP) or Efavirenz (EFZ) or Lopinavir/ritonivir-LPV/r (for those previously exposed to NVP through prevention of mother to child transmission of HIV). For children (less than 10kg in weight and/or younger than 3 years of age) with TB infection at enrolment, the preferred 1^st^ line ART choice (routinely commenced 2–8 weeks after anti-tuberculous therapy) was a combination of triple nucleoside reverse transcriptase inhibitors (NRTIs) including Zidovudine (AZT) plus 3TC plus Abacavir (ABC). AZT or D4T plus 3TC plus EFZ were prescribed for children with TB who were ≥ 10 kg in weight or older than 3 years of age. For children who developed Incident TB whilst on 1^st^ line ART and who were previously on NVP based regimen and less than 3 years of age or weighs less than 10 kg, the ART regimen was changed to triple NRTIs as stated earlier or the NVP was continued but increased to the maximum dose of 200mg/m^2^. For patients who were ≥ 3 years or ≥ 10 kg who developed incident TB, EFV is continued or the NVP is substituted with EFV. If the children were on LPV/r based regimen, the anti-tuberculous regimen would comprise rifabutin instead of Rifampicin.

Treatment failure on 1^st^ line ART was only considered after the children had been on it for at least 24 weeks, before switching to a 2^nd^ line regimen is done. For the regimen containing AZT or D4T plus 3TC plus NVP or EFV, the 2^nd^ line regimen comprised ABC or Didanosine (DDI) plus 3TC plus LPV/r and the anti-tuberculous regimen consisted of rifabutin instead of Rifampicin. For children who had LPV/r as a 1^st^ line ARV, the 2^nd^ line ART comprised one new NRTI plus 3TC plus NVP or EFV or triple NRTIs. For subjects on triple NRTIs as 1^st^ line, the 2^nd^ line regimen contained at least one new NRTI plus 3TC plus NVP or EFV or LPV/r.

Standard TB treatment comprised of Isoniazid (INH), Ethambutol (E), Rifampicin (RMP) and Pyrazinamide (Z) for 2 months, followed by INH and RMP for 4 months for pulmonary tuberculosis and 7 months for extra-pulmonary tuberculosis. For TB Re-treatment, the 8-months treatment regimen, consisted of 2 months of streptomycin [S], INH, E, RMP and Z; 1 month of RMP, INH, E and Z; and 5 months of RMP, INH and E.

### Tuberculosis Screening, Diagnosis and Definitions

#### Tuberculosis Screening

On enrolment, HIV infected children were routinely screened for tuberculosis. Screening for tuberculosis also took place during scheduled and event triggered visits. At enrolment, routine screening involved taking history suggestive of TB, a thorough physical examination and investigations which included chest radiographic imaging and tuberculin skin test (TST). In addition, when symptoms/signs suggested it, lumbar radiographic imaging, abdominal ultrasound scan, Ziehl-Neelsen staining for acid-fast bacilli (AFB) of sputum, gastric washing or cerebrospinal fluid and biopsy specimens of lymph nodes were done, at enrolment, subsequent scheduled visits and event triggered visits. The symptoms and signs (persisting for more than 2 or 3weeks) that suggested TB conformed to the ones in the *Desk-guide for diagnosis and management of TB in children* [27].

#### Tuberculosis Diagnosis

Since the confirmation of *Mycobacterium tuberculosis* using conventional culture and molecular diagnostic methods were not available in our setting; our clinical diagnosis of TB was also in keeping with the clinical TB diagnosis described by Oladokun *et al*. [28]. TB cases included children with clinical signs or symptoms suggestive of TB and any one or more of the following [28]:

1. Demonstration of the acid fast bacilli in direct smears of sputum or gastric washings.
2. Chest radiograph (CXR) consistent with pulmonary tuberculosis disease.
3. Positive clinical response to the standard regimen of 4 drugs (rifampicin, isoniazid, ethambutol and pyrazinamide for 2 months) during the intensive treatment phase of anti-tuberculosis therapy.
4. Documented exposure to a household or close contact with a tuberculosis case.
5. Positive Tuberculin Skin Test (TST) -an induration size of ≥ 5 mm (or ≥ 10 mm for subjects with a Bacillus Calmette-Guerin scar).
6. Symptoms and radiologic abnormalities suggestive of pneumonia but no response to a 10–14 day course of usual antibiotics including cotrimoxazole for Pneumocystis (further evaluated for pulmonary tuberculosis).
7. Granulomatous lesions with caseous necrosis found on histological examination of aspirate or biopsy of lymph nodes.

#### Definitions

Children on TB treatment or those diagnosed with TB at the time of enrolment into care and before ART initiation were classified as prevalent TB cases.

Incident TB was defined as the first episode of TB disease occurring after initiation of ART and categorized as early incident TB-EITB (within first 6 months of ART) or late incident TB-LITB (after 6 months in a 12 months of follow-up)-[10,29].

History of TB in a child was defined as TB that had been treated 9 months before prevalent TB and this was considered as a risk factor for both prevalent TB and Incident TB.

TB immune reconstitution inflammatory syndrome (IRIS) was defined as incident TB occurring within the first 6 months of ART, if associated with good immunological recovery and viral suppression [25, 26].

Treatment failures for HIV were considered in children who had received ART for at least 24 weeks, with ensured adherence to therapy and adequate nutrition [25, 26].

Virological failure was defined as the HIV RNA becoming reproducibly detectable again after being “undetectable” (i.e., HIV RNA PCR ˂200 copies/ml) or HIV RNA not suppressed to undetectable levels after 6 months of therapy [25, 26].

Immunological failure was defined as the return in CD4 count to pre-therapy baseline or below, in the absence of other concurrent infection to explain the transient CD4 decrease, or a greater than 50% fall from peak levels of therapy of CD4 count in the absence of other concurrent infection to explain the transient CD4 decrease [25, 26].

Clinical failure was defined as lack of growth among children who show an initial response to treatment, or a decline in growth among children who show an initial growth response to therapy, or a loss of neuro-developmental milestones or development of encephalopathy, or occurrence of new opportunistic infection or malignancy signifying clinical disease progression, or recurrence of prior opportunistic infections, such as oral candidiasis that was refractory to treatment [25, 26].

Severe immunosuppression was defined in accordance with the WHO age-dependent absolute CD4 count as follows; ˂ 1500 cell/mm^3^ in children less than 12 months, ˂ 750 cell/mm^3^ for children 12–35 months, ˂ 350 cell/mm^3^ for children 36–59 months and ˂ 200 cell/mm^3^ for those ≥ 59 months [25].

Loss to Follow- up (LTFP) was defined as an instance where the child was not seen for three consecutive months from a scheduled visit date [24].

To define under-nutrition in children less than 5 years, the weight for height z-score less than -2 standard deviations (SD) from WHO reference median was computed using the WHO Anthro software (version 2.0, 2008) which was based on WHO child growth standards of 2006 [30].

For children ≥ 5 years, the Body Mass Index (BMI) was calculated as the weight in kilograms divided by the square of the height in meters (kg/m^2^). Values ˂ 18.5 defined under-nutrition, > 25 was overweight and > 30 was obesity [31].

Sepsis in children conformed with its description including the presence of two or more of the following: abnormal temperature (˂ 36.0°C or >38.3°C) or age specific tachycardia (>140 beat/min for 0 to 2 years, >120 for 2 to 6 years and >110 for >6 years) or acute altered mental status; with a clinical suspicion of new infection including, cough/chest pain and or abdominal pain/distension/diarrhoea and/or dysuria and or headache with neck stiffness and/or presence of cellulitis/wound infection/joint infection [32]. All cases of sepsis were also confirmed with blood culture and were only counted as a separate clinical diagnosis in the absence of tuberculosis.

### Outcome of Tuberculosis Treatment

As previously categorized by Walters et al [9], the outcome of anti-tuberculous treatment for each TB episode was as follows: (1). Treatment completed and child well, where the response to treatment was good; (2). Improvement, where some symptoms persisted but the child was assessed as clinically better than at TB diagnosis; (3). No improvement, where the original symptoms persisted or worsened and (4). Death, if child died before completing TB treatment and death was linked to TB. For the third outcome, drug resistant TB could not be determined because of the unavailability of the required diagnostics and children were therefore placed on re-treatment anti-tuberculous regimen as described previously.

### Data extraction

Data were from both electronic databases and Patients’ Record Files (PRF) of the children. Missing data in the PRF were sought from the electronic databases and vice versa. A study proforma was developed to capture information that had been recorded on the children Initial Clinical Evaluation Form (ICEF) at enrollment. This includes, age, gender, place of residence (rural versus urban), number of people living in the household, history of TB, HIV/AIDS, anthropometric measurements and other diagnosed co-morbidities/opportunistic infections. These co-morbidities/opportunistic infections include, oropharyngeal candidiasis, diarrheal disease, sepsis, pneumonia, anaemia, hepatitis B and hepatitis C viral infections. Follow-up Clinical Evaluation Forms (FCEF) of the children were studied at 6 months of follow-up of each patient, to capture the following time-dependent factors including the CD4 counts, the viral load, the haemoglobin value and the anthropometric growth parameters. Information on prevalent TB, EITB, LITB, vital status state (dead or alive), children that were lost to follow-up and those who obtained transfers to other health facilities, were cumulatively sought for, up to the 6th and the 12th months of follow-up.

### Statistical analysis

Age of children in months was stratified into 4 groups (˂ 12, 12–35, 36–59, ˃ 59) in order to take advantage of the WHO age-dependent immunological grading for HIV-infected children [25]. PrevTB at the initiation of ART was determined by proportion of children having TB at enrollment. The main outcome of the study was diagnosis of TB following the commencement of ART. EITB and LITB incidence rates were calculated per 100 person years at risk (py). Censoring occurred for children that were lost to follow-up, children that were transferred out to other ART programmes, children that died and those that were followed-up to the end of the study (December 2013)

The person-time that accrued during the 6–9 months treatment for PrevTB was excluded from the denominator while calculating the EITB and LITB rates. Associations of baseline factors (socio-demographic, CD4 counts, viral load, under-nutrition, co-morbidities) with PrevTB and EITB were tested for using Logistic and Cox regression analysis respectively. Cox regression analysis was also used to assess the association between baseline factors (as noted previously), some baseline factors modified/affected by ART exposure at 6 months of follow-up [10, 33] (i.e., the CD4 count, the viral load, the haemoglobin level, anthropometry and opportunistic infections), and LITB. Variables that were significant at p -value of ≤ 0.1 in bivariate analyses were tested for at multivariate analyses. For all analyses, confidence intervals (CI) were set at 95% level and p-value less than 0.05 was considered statistically significant. Statistical analysis was done using the SPSS version 20.

## Results

**Fig 1:**
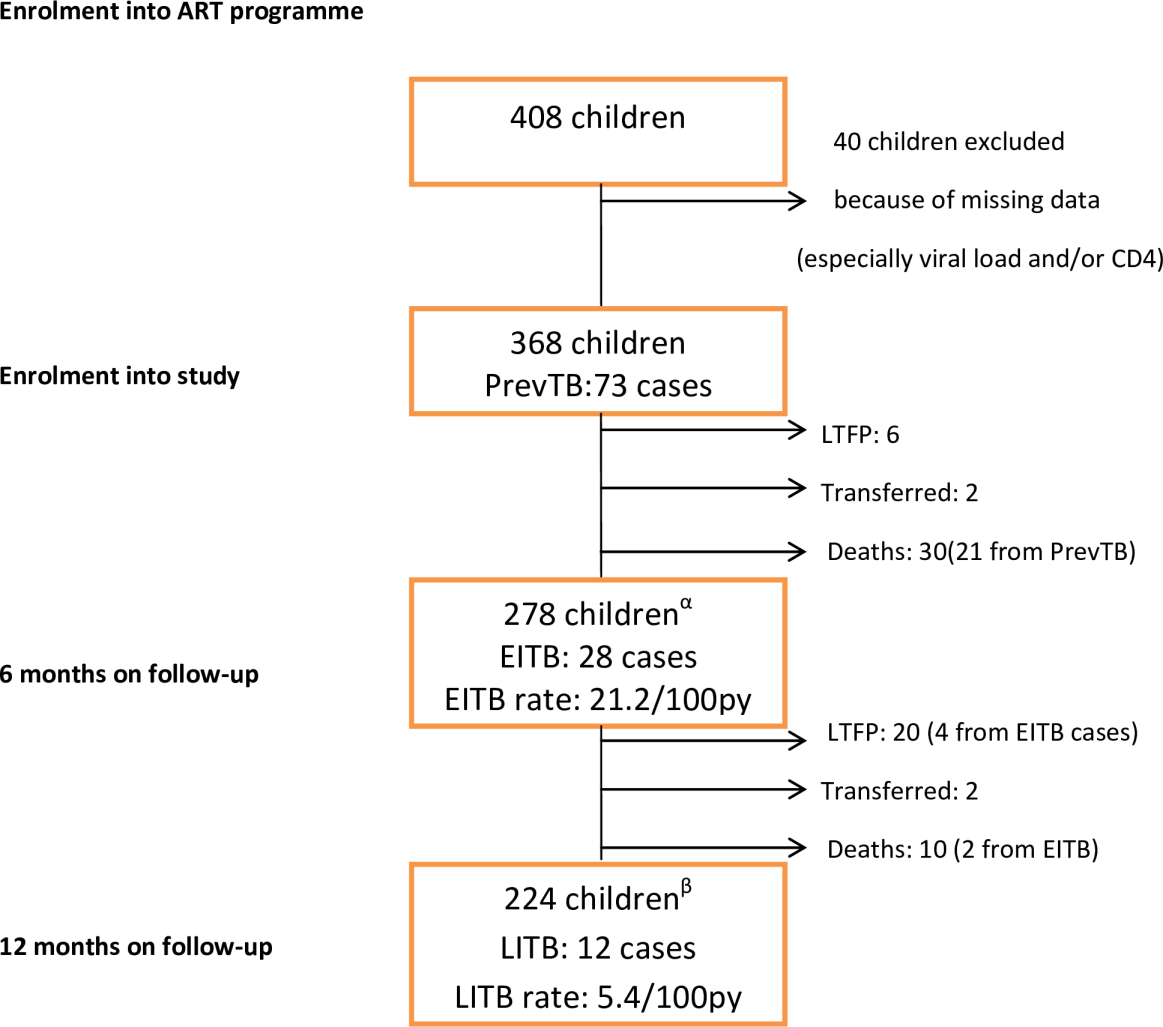
PrevTB; prevalent tuberculosis, LTFP; loss to follow-up, EITB; early incident tuberculosis, LITB; late incident tuberculosis, α; children at risk of EITB after accounting for some 90 children (i.e., 73 PrevTB cases, 6 LTFP, 2 Transfers and 9 Deaths), β; children at risk of LITB after accounting for some 54 children (i.e., 28 EITB cases, 16 LTFP, 2 Transfers and 8 Deaths), 100py; 100 person-years.

A total of 408 children were seen during the study period but only 368 were studied. Forty children (9.8%) were excluded from the study because of incomplete data and they did not differ significantly in baseline characteristics from the cohort that was studied. Only 2 of the 40 children excluded had PrevTB. Fig 1 is the schematic diagram of the follow-up of the subjects.

The age range of the 368 children studied, was from 0.3 to 13 years, with a median age of 5.63 years and interquartile range (IQR); 3–8 years. There were 206 males (M) and 162 females (F) with a M:F ratio of 1:0.8. The median CD4 count was 623.0 cells/mm^3^, IQR; 205–1067 cellmm^3^ and the median viral load (log_10_) was 4.28 (1.32) copies/ml, IQR; 3.79–5.11 copies/ml.

**Table 1**demonstrates the predictors of PrevTB among the patients at enrollment. The prevalence of TB at enrolment was 19.8% (73/368). In multivariate analyses, the age of the children, past history of tuberculosis in the children, severe immunosuppression and oropharyngeal candidiasis were significantly associated with PrevTB. Compared to older children (˃ 59 months), those in the age group of 12–35 months were 24 times more likely to have TB (95% CI; 4.1–146.6, p-value ˂0.001) and those slightly older (36–59 months) were 21 times as likely to acquire TB (95% CI; 4.0–114.3, p-value ˂0.001). Children with a past history of TB were 30 times as likely to have TB as children without history of TB (95% CI; 7.3–119.4, p-value˂0.001). HIV-infected children with severe immunosuppression were significantly associated with TB-HIV co-infection with the odds of co-infection increased by 38, compared to those with mild to moderate immunosuppression (aOR; 38.4, 95% CI; 12.0–123.2, p˂0.001). Lastly, co-infection with oropharyngeal candidiasis (aOR; 3.3, 95% CI; 1.4–8.0, p = 0.009) and sepsis (aOR; 3.2, 95% CI;1.0–9.6, p = 0.043) were also independent risk factors of PrevTB. Oropharyngeal candidiasis (p˂0.001) and sepsis (p = 0.042) also remained significant risk factors of PrevTB after controlling for viral load and CD4 counts in the regression models.

**Table 1:** Risk factors of prevalent Tuberculosis among the children at enrollment into the study.

Clinical Variables	TB* (n = 73)	No TB (n = 295)	Bivariate Logistic Regression			Multivariate Logistic Regression				
			cOR	95% CI	P -value	aOR	95% CI		P-value	
***Baseline Demography***										
**Age group (months)**										
**˂12**	8 (11.0)	24 (8.1)	4.7	1.8–12.3	0.002	5.6	0.9–37.4		0.074	
**12–35**	29 (39.7)	29 (9.8)	14.0	6.6–29.6	˂0.001	24.6	4.1–146.6		˂0.001	
**36–59**	22 (30.1)	46 (15.6)	6.7	3.2–14.1	˂0.001	21.4	4.0–114.3		˂0.001	
**>59 (Ref)**	14 (19.2)	196 (66.4)								
**Gender**										
**Female**	33 (45.2)	129 (43.7)	1.1	0.6–1.8	0.820					
**Male (Ref)**	40 (54.8)	166 (56.3)								
***Baseline Family/Socio-economic factors***										
**History of TB**										
**Yes**	32 (43.8)	32 (10.8)	6.4	3.6–11.6	˂0.001	29.5	7.3–119.4		˂0.001	
**No (Ref)**	41 (56.2)	263 (89.2)								
**Number of people living in the household**										
**>5**	50 (68.5)	146 (49.5)	2.2	1.3–3.8	0.004	1.4	0.5–4.0		0.584	
**≤5 (Ref)**	23 (31.5)	149 (50.5)								
**Place of residence of the child**										
**Urban**	44 (60.3)	126 (42.7)	2.0	1.2–3.4	0.008	1.6	0.5–4.6		0.429	
**Rural (Ref)**	29 (39.7)	169 (57.3)								
***Baseline Clinical/laboratory findings***										
**CD4 counts(cells/mm3)**										
**#Severe Immunosuppression**										
**Yes**	64 (87.7)	57 (19.3)	29.7	14.0–63.2	˂0.001	38.4		12.0–123.2	˂0.001	
**No (Ref)**	9 (12.3)	238 (80.7)								
**Viral load (copies/ml)**										
**> 10,000**	45 (61.6)	168 (56.9)	1.2	0.7–2.1	0.467					
**≤ 10,000 (Ref)**	28 (38.4)	127 (43.1)								
**Anaemia (˂8g/dl)**										
**Yes**	14 (19.2)	50 (16.9)	1.2	0.6–2.2	0.653					
**No (Ref)**	59 (80.8)	245 (83.1)								
**Hepatitis B surface antigen**										
**Yes**	2 (2.7)	38 (12.9)	0.2	0.1–0.8	0.025	0.8		0.1–6.2	0.795	
**No (Ref)**	71 (97.3)	257 (87.1)								
**Hepatitis C antibodies**										
**Yes**	2 (2.7)	6 (2.0)	1.4	0.3–6.9	0.712					
**No (Ref)**	71 (97.3)	289 (98.0)								
***Baseline co-morbidities / opportunistic infections***										
**Oropharyngeal candidiasis+**										
**Yes**	43 (58.9)	67 (22.7)	4.9	2.8–8.4	˂0.001	3.3		1.4–8.0	0.009	
**No (Ref)**	30 (41.1)	228 (77.3)								
**Diarrhoeal disease**										
**Yes**	34 (46.6)	100 (33.9)	1.7	1.0–2.9	0.045	0.8		0.3–2.0	0.593	
**No (Ref)**	39 (53.4)	195 (66.1)								
**Sepsis+**										
**Yes**	18 (24.7)	38 (12.9)	2.2	1.2–4.2	0.014	3.2		1.0–9.6	0.043	
**No (Ref)**	55 (75.3)	257 (87.1)								
**Pneumonia**										
**Yes**	11 (15.1)	47 (15.9)	0.9	0.5–1.9	0.856					
**No (Ref)**	62 (84.9)	248 (84.1)								
**WHZ**										
**<-2 SD**	15 (25.4)	25 (25.3)	1.0	0.5–2.1	0.981					
**≥-2 SD (Ref)**	44 (74.6)	74 (74.7)								
**BMI**										
**<18.5**	10 (71.4)	160 (81.6)	0.6	0.2–1.9	0.353					
**≥18.5 (Ref)**	4 (28.6)	36 (18.4)								

**T**B = tuberculosis

* = 73 cases of TB, giving the proportion prevalence of TB of 19.8% (73/368); + = sepsis(p = 0.042) and oropharyngeal candidiasis (p˂0.001) were significant risk factors after controlling for viral load and CD4 counts in the regression models; WHO = World Health Organization;# = 2006 WHO age-dependent immunological criteria dichotomized into Yes or No for severe immunosuppression; WHZ = weight for health Z score; BMI = body mass index; Ref = reference group

**Table 2**shows the risk factors for EITB following the commencement of ART. The cumulative exposure time between enrolment into the programme and ART initiation was 50.931yrs with a median time of 0.08 years. In the first 6 months of starting ART, 28 EITB cases were recorded among 278 children, over 132 person-years (py) with an EITB rate of 21.2/100 py (95% CI; 14.1–30.7). Out of the 28 cases, only 2 (7.1%) were in keeping with TB IRIS. The median time to developing the EITB was 3.1months (Interquartile range of 2.04–4.09 months). In the unadjusted Cox regression analyses, children within the age group of 12–35 months were more likely to have EITB (cHR; 3.5, 95% CI; 1.6–8.0,p = 0.003), whereas, those with urban residency (cHR; 0.1, 95% CI;0.0–0.4, p = 0.001) were at significant reduced risk of having EITB. The risk of EITB did not depend on the regimens of ART or on the severity of immunosuppression. However, in multivariate analyses, only urban residency was found to be associated with reduced risk of EITB (aHR; 0.1, 95% CI; 0.0–0.4, p = 0.001).

**Table 2:** Risk factors of Early Incident Tuberculosis following antiretroviral therapy.

Clinical Variables			EITB* (n = 28)	No EITB (n = 250)	Bivariate Cox Regression			Multivariate Cox Regression		
					cHR	95% CI	P–value	aHR	95% CI	P-value
***Baseline Demography***								
**Age group (Months)**								
**˂12**	0 (0)	18 (7.2)	0.0		0.978	0.0		0.973
**12–35**	8 (28.6)	16 (6.4)	3.5	1.6–8.0	0.003	1.9	0.8–4.7	0.173
**36–59**	0 (0)	44 (17.6)	0.0		0.965	0.0		0.960
**>59 (Ref)**	20 (71.4)	172 (68.8)						
**Gender**								
**Female**	10 (35.7)	116 (46.4)	0.7	0.3–1.4	0.302			
**Male (Ref)**	18(64.3)	134 (53.6)						
**Types of HAART on enrolment**								
**LPV/r based**	0 (0)	0 (0)						
**ABC/3TC/AZT or d4T**	0 (0)	4 (1.6)	0.1	0.0–37622.4	0.662			
**NNRTI based (Ref)**	28 (100)	246 (98.4)						
***Baseline family/Socio-economic factors***							
**History of TB**								
**Yes**	6 (21.4)	26 (10.4)	2.3	0.9–5.6	0.076	2.1	0.8–5.3	0.134
**No (Ref)**	22 (78.6)	224 (89.6)						
**Number of people living in the household**								
**>5**	12 (42.9)	126 (50.4)	0.8	0.4–1.6	0.466			
**≤5 (Ref)**	16 (57.1)	124 (49.6)						
**Place of residence of the child**								
**Urban**	2 (7.1)	118 (47.2)	0.1	0.0–0.4	0.001	0.1	0.0–0.4	0.001
**Rural (Ref)**	26 (92.9)	132 (52.8)						
***Baseline Clinical/laboratory findings***								
**CD4 counts (cells/mm**^**3**^**)**								
**#Severe Immunosuppression**								
**Yes**	6 (21.4)	40 (16.0)	1.4	0.6–3.5	0.460			
**No (Ref)**	22 (78.6)	210 (84.0)						
**Viral load (copies/ml)**								
**>10,000**	18 (64.3)	139 (55.6)	1.4	0.7–3.1	0.381			
**≤10,000(Ref)**	10 (35.7)	111 (44.4)						
**Anaemia (˂8g/dl)**								
**Yes**	4 (14.3)	42 (16.8)	0.8	0.3–2.4	0.721			
**No (Ref)**	24 (85.7)	208 (83.2)						
**Hepatitis B surface antigen**								
**Yes**	6 (21.4)	32 (12.8)	1.8	0.7–4.4	0.201			
**No (Ref)**	22 (78.6)	218 (87.2)						
**Hepatitis C antibodies**								
**Yes**	2 (7.1)	4 (1.6)	3.7	0.9–15.5	0.076	2.9	0.6–15.0	0.205
**No (Ref)**	26 (92.9)	246 (98.4)						
***Baseline co-morbidities/opportunistic infections***								
**Oropharyngeal candidiasis**								
**Yes**	4 (14.3)	58 (23.2)	0.6	0.2–1.6	0.289			
**No (Ref)**	24 (85.7)	192 (76.8)						
**Diarrhoeal disease**								
**Yes**	10 (35.7)	84 (33.6)	1.1	0.5–2.3	0.848			
**No (Ref)**	18 (64.3)	166 (66.4)						
**Sepsis**								
**Yes**	2 (7.1)	36 (14.4)	0.5	0.1–2.0	0.310			
**No (Ref)**	26 (92.9)	214 (85.6)						
**Pneumonia**								
**Yes**	8 (28.6)	36 (14.4)	2.3	1.0–5.1	0.050	2.1	0.9–4.8	0.083
**No (Ref)**	20 (71.4)	214 (85.6)						
**WHZ**								
**<-2 SD**	2 (25.0)	20 (25.6)	1.0	0.2–5.0	0.999			
**≥-2 SD (Ref)**	6 (75.0)	58 (74.4)						
**BMI**								
**<18.5**	18 (90.0)	138 (80.2)	2.1	0.5–9.2	0.308			
**≥18.5 (Ref)**	2(10.0)	34(19.8)						

EITB = early incident tuberculosis

* = 28 EITB cases were recorded among 278 children over 132 person-years (py) with an EITB rate of 21.2/100 py (95% CI; 14.1–30.7); 2 cases (7.1%) were in keeping with TB IRIS; the median time to developing the EITB among the children was 3.1months; NNRTI(Non-nucleoside reverse transcriptase inhibitors; LPV/r = lopinavir/ritonavir; ABC = Abacavir; 3TC = Lamivudine; AZT = Zidovudine; d4T = starvudine; HAART = highly active antiretroviral therapy; WHO = World Health Organization; # = 2006 WHO age-dependent immunological criteria dichotomized into Yes or No for severe immunosuppression; WHZ = weight for health Z score; BMI = body mass index.

**Table 3**reflects the risk factors of LITB cases among the children. Twelve LITB cases were seen among 224 children over 221.9 py with a cumulative LITB rate of 5.4/100 py (95% CI; 2.8–9.4) A significant drop off in rates existed between EITB and LITB (incident rate difference of 15.8/100py, 95% CI; 8.6–23.0) over time with a p value < 0.001. In other words, ART reduces the incident rates of TB by 75%, between 6 months and 12 months of follow-up. The median time to LITB after ART initiation was 10.3months.

**Table 3:** Risk factors of Late Incident Tuberculosis following antiretroviral therapy.

Clinical Variables		EITB* n = 12)	No EITB (n = 212)	Bivariate Cox Regression			Multivariate Cox Regression		
				cHR	95% CI	P -value	aHR	95% CI	P-value
***Baseline Demography ***								
**Age group (Months)**								
**˂12**	0 (0)	18 (8.5)	0.0		0.986			
**12–35**	0 (0)	12 (5.7)	0.0		0.988			
**36–59**	4 (33.3)	38 (17.9)	1.9	0.6–6.4	0.303			
**>59 (Ref)**	8 (66.7)	144 (67.9)						
**Gender**								
**Female**	6 (50.0)	114 (53.8)	1.2	0.4–3.6	0.788			
**Male (Ref)**	6 (50.0)	98 (46.2)						
***Baseline family/Socio-economic factors ***								
**Number of people living in the household**								
**>5**	8 (66.7)	100 (47.2)	2.2	0.7–7.1	0.212			
**≤5 (Ref)**	4 (33.3)	112 (52.8)						
**Place of residence of the child**								
**Urban**	6 (50.0)	96 (45.3)	1.2	0.4–3.7	0.762			
**Rural (Ref)**	6 (50.0)	116 (54.7)						
***Baseline Clinical/laboratory findings ***								
**Hepatitis B surface antigen**								
**Yes**	2 (16.7)	28 (13.2)	1.3	0.3–5.9	0.742			
**No (Ref)**	10 (83.3)	184 (86.8)						
**Hepatitis C antibodies**								
**Yes**	0 (0)	0 (0)						
**No (Ref)**	12 (100)	212 (100)						
***Laboratory responses at 6 months of HAART***								
**CD4 counts (cells/mm**^**3**^**)**								
**#Severe immunosuppression**								
**Yes**	4 (33.3)	38 (17.9)	2.2	0.7–7.4	0.191			
**No (Ref)**	8 (66.7)	174 (82.1)						
**Viral load (copies/ml)**								
**>200**	4 (33.3)	25 (11.8)	3.5	1.1–11.8	0.039	4.7	1.3–16.5	0.017
**≤ 200 (Ref)**	8 (66.7)	187 (88.2)						
**Anaemia (˂8g/dl)**								
**Yes**	0 (0)	22 (10.4)	0.04	0.0–163.2	0.453			
**No (Ref)**	12 (100)	190 (89.6)						
***Co-morbidities/opportunistic infections at 6 months of HAART ***								
**Oropharyngeal candidiasis**								
**Yes**	0 (0)	0 (0)						
**No (Ref)**	12 (100)	212 (100)						
**Diarrhoeal disease**								
**Yes**	0 (0)	12 (5.7)	0.1	0.0–2760.0	0.583			
**No (Ref)**	12 (100)	200 (94.3)						
**Sepsis+**								
**Yes**	2 (16.7)	2 (0.9)	18.1	4.0–83.2	˂0.001	26.5	5.3–131.9	˂0.001
**No (Ref)**	10 (83.3)	210 (99.1)						
**Pneumonia**								
**Yes**	0 (0)	4 (1.9)	0.1	0.0–7419106.4	0.753			
**No (Ref)**	12 (100)	208 (98.1)						
***Anthropometric responses at 6 months of HAART ***								
**WHZ**								
**<-2 SD**	0 (0)	2 (2.9)	0.0		0.988			
**≥-2 SD (Ref)**	4 (100)	66 (97.1)						
**BMI**								
**<18.5**	8 (100)	132 (91.7)	24236		0.957			
**≥18.5 (Ref)**	0 (0)	12 (8.3)						

LITB = late incident tuberculosis

* = .12 LITB cases were seen among 224 children over 221.9 py with a cumulative LITB rate of 5.4/100 py (95% CI; 2.8–9.4).A significant drop off in incident rates existed between EITB and LITB (incident rate difference of 15.8/100py (95% CI; 8.6–23.0) with a p value < 0.0001.The median time to LITB after ART initiation was 10.3months; + = sepsis (p = 0.003) remained a significant risk factor after controlling for viral load and CD4 counts in the regression models; HAART = highly active antiretroviral therapy; WHO = World Health Organization; # = 2006 WHO age-dependent immunological criteria dichotomized into Yes or No for severe immunosuppression; WHZ = weight for health Z score; BMI = body mass index.

In the adjusted Cox regression analyses, children with virological failure (viral load ˃ 200 copies/ml) were at a higher hazard of LITB (aHR; 4.7, 95% CI; 1.3–16.5, p = 0.017) than those with virological suppression. Also, children co-infected with sepsis (aHR; 26.5, 95% CI; 5.3–131.9, p ˂0.001) were at an increased risk of acquiring LITB. Furthermore, sepsis also remained a significant risk factor for LITB after controlling for viral load and CD4 counts in the regression models (p = 0.003). However, the risk of LITB did not depend on the degree of the immunosuppression (CD4 counts).

**Table 4**depicts the pattern and outcome of the Tuberculosis cases. For the 73 PrevTB cases, majority (49, 67.1%) were pulmonary TB, 9 (12.3%) had miliary TB/TB meningitis, 10 (13.7%) were TB adenitis, 3 (4.1%) had gastrointestinal TB and 2 (2.7%) had TB of the cervical vertebrae. Twenty-nine (29/49, 59.2%) of the pulmonary TB cases received treatment and got well, whereas, 22.4% (11/49) of the pulmonary TB cases died. Most (7/9, 77.8%) of the cases of the military TB/ TB meningitis died. Among the 28 EITB cases, 8 were of the miliary TB/TB meningitis and 20 were pulmonary TB. Four (50%) of the 8 cases of miliary TB/TB meningitis were transferred out to other health facilities and mortality was 25% (2 deaths). Treatment was favourable among the pulmonary TB cases as 16 (80%) of them got well. For the 12 LITB cases, 10 (83.3%) were pulmonary TB and 2 (16.7%) were TB adenitis. Half of the pulmonary TB cases got well after treatment and 1 died. One of the 2 cases of the TB adenitis got well after treatment and one was lost to follow-up.

**Table 4:** Pattern and outcome of the Tuberculosis cases.

Tuberculosis Cases	Treatment completed and child well	Clinical improvement but symptoms persist	No improvement	Death	Lost to Follow-up
**1.Prevalent TB(N = 73)**					
A. Pulmonary TB	29	6	3	11	-
N = 49					
B. TB adenitis	9	-	-	1	-
N = 10					
C. TB Meningitis/Miliary TB	-	1	1	7	-
N = 9					
D. Gastrointestinal TB	1	-	-	2	-
N = 3					
E. TB bone (thoracic vertebra)	1	1	-	-	-
N = 2					
**2.EITB (N = 28)**					
A. TB Meningitis/Miliary TB	1	1	-	2	4
N = 8					
B. Pulmonary TB	16	3	1	-	-
N = 20					
**3. LITB (N = 12)**					
	
A. Pulmonary TB	5	1	-	1	3
N = 10					
B. TB adenitis		-	-	-	1
	1				
N = 2					

TB = tuberculosis, EITB = early incident tuberculosis, LITB = late incident tuberculosis

## Discussion

With a prevalence of 19.8% of TB at ART initiation of HIV 1-infected children, the present study suggests that TB is endemic in our setting. When compared to other Nigerian studies, our prevalence was similar to the 19.5% reported in Abuja [11] and the 15.2% in Nnewi [19]; whilst it was higher than the 10.5% in Sagamu [12]; it was definitely lower than the respective 31.4% and 41.7% from Zaria [18] and Ibadan [17]. In other countries in Sub-Saharan Africa and in Europe, our prevalence was lower than the 40% in the data published by Walters *et al*. [9] from South Africa, but was higher than the respective 9.5%, 5.5% and 3.6% from Uganda [33], United Kingdom [34] and Kenya [13]. The variation in the background TB rates in the communities and the differences in diagnostic definitions and procedures may account for the different burden of TB in the different settings.

Children in the lower age groups (12–59 months) were more likely to have prevalent TB in the present study. This contrasted with the study of Okechukwu and Okechukwu [11] in Abuja, Nigeria, where, significantly more TB cases were seen among children older than 5 years. Immaturity of the immune system, coupled with a poor cell-mediated immunity and the consequent unrestrained mycobacterial proliferation has been postulated to be responsible for a high rate of TB in young children [7, 35]. However, the increased cumulative probability of exposure to adults with smear positive TB in TB endemic communities as children gets older, is a contrariwise risk factor, and may also explain the finding of Okechukwu and Okechukwu [11].

A prior history of TB in the children also increased the risk of PrevTB in this study. However, we could not prove whether the PrevTB cases seen at ART enrollment were cases of relapse, re-infection or multidrug resistant TB as the necessary diagnostics required to make the distinction among the three possibilities were unavailable in our setting. Similar finding had been reported earlier among children [8, 9] and adult populations [34,35]. Within the context of HIV, relapse may vary according to the anti-tuberculous regimens and the prevalence of drug resistant strains [35]. Higher relapse rate of TB in HIV is also expected as effective tuberculous chemotherapy requires the support of a functioning immune system which is compromised with HIV infection [36]. Re-infection varies according to the background risk of TB in the environment as HIV also increases the risk of re-infection via an increased risk of exposure to TB cases at ART clinics [35, 37].

Severe immunosuppression also increased the risk of PrevTB in this study. In paediatric populations with HIV, similar finding had been reported by Okechukwu and Okechukwu [11], Walters *et al*. [9], Braitstein *et al*. [13], and Marais *et al*. [38]. HIV infection depletes the CD4 cell counts with its attendant’s susceptibility to opportunistic infections of which TB is common. TB on itself also lowers the CD4 cell count further [16]. A defective chemotaxis, defective granuloma formation and maintenance, impaired antigen processing and presentation, selective clonal depletion of TB specific CD4+ lymphocytes [16,39], and decreased apoptosis of TB infected alveolar macrophages also explain the risk of TB acquisition and progression in HIV associated immune dysfunctions[16,39].

Oropharyngeal candidiasis and sepsis were other risk factors of PrevTB in the present study. Agbaji and co-workers had also reported a similar association of oropharyngeal candidiasis and TB among HIV-infected adults in Jos, Nigeria [40]. It is also important to note that oropharyngeal candidiasis and sepsis also remained as independent risk factors, even after controlling for CD4 counts and viral load.

Twenty-eight EITB cases were seen in the first 6 months of ART, with an EITB rate of 21.2/100 py. After the first 6 months of ART, a lower 12 LITB cases were seen, with a cumulative LITB rate of 5.4/100 py. It is therefore noteworthy that, a significant drop off in rates existed between EITB and LITB over time, with a reduction as high as 75%.

Similarly, in other paediatric cohorts on ART, Martinson *et al*. [14] in South Africa, Li *et al*. [41] in Tanzania and Bakeera-Kitaka *et al*. [33] in Uganda, had previously reported a reduction in incident TB by 70% (Braitstein *et al*. by 85%[13]), thereby, underscoring the importance of ART in the prevention of incident TB among HIV-infected children. However, whilst a reduction of incident TB cases has been shown over 7 years of ART in the study of Mu *et al*. [42] in China, contrariwise, Ayalaw *et al*. [43] documented a high incident TB cases over 6 years of ART in Ethiopia.

Also, the reduction in the incident TB cases by ART in this cohort, took place regardless of the degree of immunosuppression at 6 months of ART. This finding agrees with the review of Suthar *et al*. in adult populations, where, ART was strongly associated with a reduction in TB incidence across all CD4 cell counts levels [44].

In the present study, TB IRIS could only explain two of the 28 (7.1%) reported cases of EITB. Our finding tends to support the rarity of TB IRIS in paediatric populations, in keeping with the data of Okechukwu and Okechukwu-4.9% [11] and those of Walters *et al*.*-*7.4% [9]. The remaining 26 (92.9%) EITB cases could be PrevTB cases that were missed at enrolment into our ART programme, even with our keen attempts at TB case finding among our new enrolees. However, this is not surprising as HIV infection is well known for its ability to reduce the sensitivity and the specificity of the screening symptoms and signs of TB [16]. A possibility of missed PrevTB cases is also strengthened by the fact that we made use of clinical diagnosis of TB cases in this study and also because of the rather short median time (3.1 months) to EITB diagnosis.

Urban residency was found to be associated with reduced risk of EITB. This is an unusual finding as the risk of TB is expected to increase with overcrowding that is more prevalent in urban households. On the other hand, the exposure of household to air population from firewood smoke [11,45], a well-known host risk factor for progression of TB infection to TB disease, is uncommon in urban setting and may partly explain this protection against EITB. In addition, a higher awareness/education about how to protect children from contracting TB from a suspected tuberculosis cases may be more prevalent in urban setting and could also explain this finding.

For LITB, sepsis was the strongest risk factor. Similar to being a risk factor of PrevTB, sepsis also remained an independent risk factor for LITB, even after controlling for the CD4 counts and viral load and children who developed sepsis on ART should be closely screened for TB. Both ART-naïve and ART experienced subjects have a significantly increased risk of developing sepsis [46] and a combination of factors might explain the increased susceptibility to infection in this patient group [46]. For instance, HIV-induced immune perturbations, low CD4/CD8 ratio and the residual immune dysregulation syndrome are some of the factors that had been described previously in the pathogenesis of HIV-associated sepsis [46]. In addition, sepsis also causes a number of defects in immune function, including a shift from a pro-inflammatory (Th1) to an anti-inflammatory (Th2) cytokine profile, increased production of the anti-inflammatory cytokine IL-10, monocyte deactivation with low HLA-DR expression, and apoptosis of B and CD4 T lymphocytes [47, 48].

Not surprisingly, children with virological failure at 6 months of ART were also found to be at risk of LITB. Virological failure increases the risk of acquiring opportunistic infection of which TB is one. This finding however, contrasted the United States and European Antiretroviral Therapy Cohort Collaboration study, whereby, adult subjects without virological suppression were 2 times more at risk of TB [49].

## Conclusion

In our setting, HIV-TB co-infection is common before ART and the risk of incident TB remained in the first 6 months of ART but decreased significantly thereafter through the first year. At enrollment into our ART programme, a high index of suspicion is required for early TB diagnosis among young HIV-infected children (12–59 months), those with prior history of TB, and children who presented with severe immunosuppression, oropharyngeal candidiasis and sepsis. After the commencement of ART, children of rural residency and those with sepsis and virological failures should also be intensively screened for incident TB.

## Limitation of study

Being a retrospective cohort study, it is limited by incomplete and missing data. Also, the sample size was drawn from a tertiary health facility and as such, cases of PrevTB and the incident TB reported may have been higher than the general paediatric population. Contrariwise, there may have been under-diagnosis of TB because of the well-known incomplete ascertainment of TB (even presumptive TB) in children. Over-diagnosis may also be possible because we made use of clinical case diagnosis of TB. In addition, a relapse or a re-infection or a multidrug resistant TB could not be differentiated because of unavailability of resistant testing and molecular diagnostics in our center. Furthermore, a longer ART follow-up period of more than 1 year could have been more appropriate for clarifying the impact of ART on incident TB. However, an adequate sample size and the fact that time-dependent co-founders were accounted for in the assessment of the risk of LITB strengthened this study.
